# After War, Armistice. After Armistice, Peace?

**Published:** 1919-12-27

**Authors:** 


### The Hospital

The Workers' Newspaper of Administrative Medicine and Institutional Life, Administration, National Insurance and Health.

No. 1751, Vol. LXVII. SATURDAY, DECEMBER 27, 1919. PRICE TWOPENCE

### AFTER WAR, ARMISTICE. AFTER ARMISTICE, PEACE?

The Armistice was signed on November 11, 1918. More than a year has elapsed, and peace has still to be attained. Everything possible has been done by the European Allies and the Dominions to secure the termination of hostilities and the establishment of peace throughout the world. The position as it stands to-day is none of their seeking. It is a disappointment to the world, but optimism is still in possession of the peoples, and it says much for the common-sense of the nations, outside those which at present block the way, that the world should have been so united in opinion, and have such faith in the righteousness of the claims which peace properly makes and which the consummation of peace must enforce. The sooner this happens the better will it be for all the peoples of the world. That few or none can doubt, and it is an amazing contretemps that party politicians—who for the most part will probably find no place in history, if indeed any of them will—should dilly-dally with the gravest issues, and block the way with deliberate intent.

It is no part of our intention or duty either to read a lesson or to give expression to views. Our duty to-day is simply to record the facts, as illustrated by the position, to pass on to examine the circumstances of the moment, and take courage for the future. We in England have the privilege of already welcoming back a large proportion of our warriors and notable women workers, who in hundreds of thousands are now with their families and friends in the Homeland. For them, one and all, Christmas, 1919, must be memorable indeed. It seems to us to be highly desirable that the delays incidental to the signing of the Peace Treaty of certain nations should not diminish by one iota family rejoicings throughout Great Britain. The glorious happiness of the deliverance which the war has brought, the honour of and thanksgivings for those brave men and women who have given their lives in the cause of their country and the nation's security, should have full expression without delay. The business of the world must proceed, and should there unfortunately prove to be some nations or people unable to make up their mind to take action of any kind, they must acknowledge the asphyxiation from which they are suffering and leave the world at large to pursue its way.

Everywhere we must have rejoicing. It is due to the British nation and Empire that they should unite and rejoice among themselves at the victory which in God's good providence has been granted to them. The war has been a wonderful educator. It has brought out qualities in individuals which were not suspected even by their most intimate friends. It has demonstrated that the robustness of the race stands where it ever did, and that the prowess, judgment, courage, and resolution of the people, individually and collectively, have awakened and won the admiration of the whole world. Christmas Day, 1919, can properly, therefore, be spent without gloomy forebodings or disappointed hopes, for time is on our side. The days of fulfilment to the victors are approaching, with rapid steps, and must bring with them the enforcement of justice, to the uplifting of all that is noblest and best, and the punishment and correction of those who have broken their faith and have fallen from their former high estate in consequence. The thirteen months and more since the Armistice have been filled with many happenings, and the granting of an amount of liberty, leisure, and pleasurable enjoyment unequalled in any other period of our history. The time thus spent is, we are glad to say, showing signs of resulting in a due apprehension of the needs of the nation which demand the speedy readjustment that will no doubt find fruition with the beginning of 1920. Then the watchword will be: "All hands to the pumps," and an awakened and rested people will, we believe, put their backs into the work with a will, which will speedily bring the business of the nation on to firm ground. Then the output of its industries should speedily reach a yield and results never exceeded in our history.

With such prospects the British people can turn this week with light hearts to the celebration of Christmas in a manner which will bring together all types of people, knit up the family circles, rejoice the hearts of the children, and make the land merry with the signs of rejoicing and thankfulness, for the great mercies resulting from the war. We hope the Churches and the People will join in united worship and heartfelt thanksgiving, everywhere, throughout Great Britain. Then Christmas Day, 1919, should be memorable for all time. It should mark the beginning of brighter and happier days for the multitude of the people of all classes. The awakening of the Nation and Empire to the many causes which unite to give them joyous hearts, and the steady outlook of a resolute, grateful, convinced people, in the presence of the living God, and their dependence upon Him in all things, now and always. Rightly understood and properly grasped, Christmas Day, 1919, should be made a great day indeed, in which all the people, of every class and type will join hands and rejoice greatly, that they have thus passed, safely, through the most awful testing and arduous days which have ever befallen our race. Victory is indeed ours, but its possession renders us responsible for the future, because the making of the future, in God's good Providence, depends so largely upon each individual man and woman. Each will have the privilege of taking part in all the great happenings which are beginning to unfold to their vision, and to demonstrate the greatness of the opportunity, and the necessity, as well as the glory, of unity. For unity must mean the assured certainty, that if we faint not but believe, England will grow greater and greater, her powers for good must ever extend, and the lustre which attaches to her history become brighter and wider, as the days pass and the years increase.

